# MODERATING EFFECT OF ANXIETY ON PROACTIVE CONTROL OF EMOTION ACROSS AGE

**DOI:** 10.1093/geroni/igad104.3478

**Published:** 2023-12-21

**Authors:** Ziyuan Chen, Bruna Martins-Klein, Ava Varu

**Affiliations:** University of Southern California, Los Angeles, California, United States; University of Southern California, Los Angeles, California, United States; University of Southern California, Los Angeles, California, United States

## Abstract

Anxiety and aging predict difficulties engaging cognitive control and emotion regulation strategies like reappraisal. Dual mechanisms of control (DMC) theory suggests that cognitive control is engaged in anticipation of a task (proactive control) or in-the-moment (reactive control). Both aging and anxiety predict limited benefit from proactive control and greater reliance on reactive control during cognitive tasks. However, this remains unexamined in the emotion regulation context. In this eyetracking study, 41 younger (18-29) and 40 older adults (60-85) were cued to reappraise or view 80 negative images after a proactive (4s) or reactive delay (500 ms) prior to image onset, as regulatory arousal and effort were tracked by pupil responsivity. Multilevel models revealed that anxiety was associated with greater benefit of proactive over reactive control (reduced pupil response) for older adults during reappraisal and viewing (p < .001). For younger adults, anxiety was associated with reduced benefits of proactive compared to reactive reappraisal use. Image viewing in younger adults revealed an interaction, where low anxiety predicted greater benefit from reactive control, and moderate to highly anxiety predicted greater benefit from proactive control. Thus, while older age was associated with greater proactive benefits in both emotional reactivity and reappraisal, younger adults only showed proactive control benefits during image viewing when anxiety was elevated. Future work should explore the clinical benefit of proactive training in late-life anxiety, and how emotional reactivity and reappraisal outcomes differ with cognitive control temporal dynamics in individuals with diagnosed anxiety disorders across the lifespan.

